# Correction: ‘Thermal efficiency extends distance and variety for honey bee foragers: analysis of the energetics of nectar collection and dessication by *Apis mellifera*’ (2019), by Derek Mitchell

**DOI:** 10.1098/rsif.2023.0598

**Published:** 2023-11-15

**Authors:** Derek Mitchell

**Affiliations:** School of Mechanical Engineering, Leeds University, Leeds Yorkshire, UK

**Keywords:** evaporation, nectar, efficiency, ripening, climate

*J. R. Soc. Interface* **16**, 20180879 (Published online 23 January 2019). (doi:10.1098/rsif.2018.0879)

This article corrects the following:

Errors were found in table 2 [1] in the size of entrances, heat capacity of air and also in the quoted lumped conductances of hives and trees in the introduction. This led to the following amendments to the introduction and table 2. All other results and the conclusions drawn are unaffected.

**Table 2.** General parameters.

item	value	source	item	value	source	item	value	source
*κ*_Air_	1.2 kJ K^−1^ kg^−1^	[58]	*ρ*_Air_	1 kg m^−3^	[58]	*A*_Entrance_	10 × 10^−4^ m^2^	[60]
*L*_Sucrose_	16.2 MJ kg^−1^	[59]	*u*_Entrance_	0.94 m s^−1^	[49]	*C*_Honey_	0.8	[4,45]
*φ*	162.5 J kg^−1^ m^−1^	[4]	*L*_Water_	2.426 MJ kg^−1^ @305 K	[7]			

Typical values for the lumped conductance range from 1 W K^−1^ for tree nests to 3 W K^−1^ for man-made nests. Typical values for entrance size and fanned air velocity are 10 cm^3^ [4] and 1 m s^−1^ [49]. These give an advection term of around 0.5 W K^−1^; thus for hives we can ignore the energy in the advection caused by honey bees fanning at the entrance.

This has been corrected on the publisher's website.

## References

[1] Mitchell DM. 2019 Thermal efficiency extends distance and variety for honeybee foragers: analysis of the energetics of nectar collection and desiccation by *Apis mellifera*. J. R. Soc. Interface **16**, 20180879. (10.1098/rsif.2018.0879) 30958150 PMC6364643

